# Surgical Options for Aortic Root Replacement in Destructive Endocarditis

**DOI:** 10.21470/1678-9741-2020-0020

**Published:** 2020

**Authors:** Marcin Szczechowicz, Alexander Weymann, Sabreen Mkalaluh, Ahmed Mashhour, Konstantin Zhigalov, Jerry Easo

**Affiliations:** 1 Department of Cardiac Surgery, Oldenburg University Hospital, Oldenburg, Germany.

**Keywords:** Endocarditis, Bacterial, Aortic Valve, Reoperation, Heterografts, Transplantation, Heterologous, Stents, Allografts

## Abstract

**Objective:**

To analyze patients’ preoperative characteristics, surgical data, postoperative courses, and short- and long-term outcomes after implantation of different full-root prostheses for destructive aortic valve endocarditis.

**Methods:**

Between 1999 and 2018, 80 patients underwent aortic root replacement due to infective endocarditis in our institution. We analyzed the abovementioned data with standard statistical methods.

**Results:**

The Freestyle stentless porcine prostheses were implanted in 53 (66.25%) patients, biological valve conduits in 13 (16.25%), aortic root homografts in nine (11.25%), and mechanical valve conduits in five (6.25%). There were no significant preoperative differences between the groups. The incidence of postoperative complications and intensive care unit length of stay did not differ significantly between the groups. The 30-day mortality rate was low among Freestyle patients (n=8, 15.1%) and high in the mechanical conduit cohort (n=3, 60%), though with borderline statistical significance (*P*=0.055). The best mean survival rates were observed after homograft (13.7 years) and stentless prosthesis (8.1 years) implantation, followed by biological (2.8 years) and mechanical (1.4 years) conduits (*P*=0.014). The incidence of reoperations was low in the mechanical conduit group (0) and stentless bioroot group (n=1, 1.9%), but two (15.4%) patients with biological conduits and three (33.3%) patients with homografts required reoperations in the investigated follow-up period (*P*=0.005).

**Conclusion:**

In patients with the destructive form of aortic valve endocarditis, homografts and stentless porcine xenografts offer better survival rates than stented valve conduits; however, the reoperation rate among patients who received homograft valves is high.

**Table:** 

Abbreviations, acronyms & symbols	
**ECMO**	**= Extracorporeal membrane oxygenation**
**EuroSCORE**	**= European System for Cardiac Operative Risk Evaluation**
**FFCE**	**= Freedom from combined endpoint**
**IABPs**	**= Intra-aortic balloon pumps**
**IE**	**= Infective endocarditis**
**NVE**	**= Native valve endocarditis**
**PVE**	**= Prosthetic valve endocarditis**

## INTRODUCTION

Infective endocarditis (IE), if left untreated, is almost always lethal. In the pre-antibiotic era, most IE patients died due to sepsis, often before congestive heart failure caused by valve destruction could occur^[1]^. Today, the incidence of IE remains unchanged and amounts to 30 to 100 per million patient-years^[2]^. Even in these times of prophylactic treatment, modern antimicrobial therapy, advanced surgical methods, and structured guidelines, up to 30% of IE patients still die within the first year after the diagnosis^[3]^. A devastating complication that occurs in 10-40% cases of aortic valve IE is periannular extension of the infectious process with consecutive impairment of valve function, fistula formation, obstruction of coronary arteries, severe arrhythmias, pseudoaneurysm formation, or even sudden cardiac death^[4-6]^. Immediate surgical treatment in the acute phase of infection remains the gold standard treatment^[6]^. A further high-risk cohort of patients are the 20% IE cases with prosthetic valve endocarditis (PVE). The prevalence of PVE grows steadily and the prognosis is worse than in cases of native valve endocarditis (NVE) due to the excavating destruction of periannular structures, which occurs in most cases (56% to 100%)^[7,8]^. The infection of the valve prosthesis often leads to abscess formation or detachment of the valvular ring and is associated with increased mortality^[8]^.

Surgical therapy is essential for effective and successful treatment of IE and requires clear guidelines for the optimal treatment algorithm^[9]^. In cases of destructive root endocarditis, two surgical treatment strategies have shown promising longterm results: extensive root reconstruction and root replacement. There are no clear indications for the first or the second option, but root replacement seems to be associated with lower reoperation rates^[10]^.

**Fig. 1:**
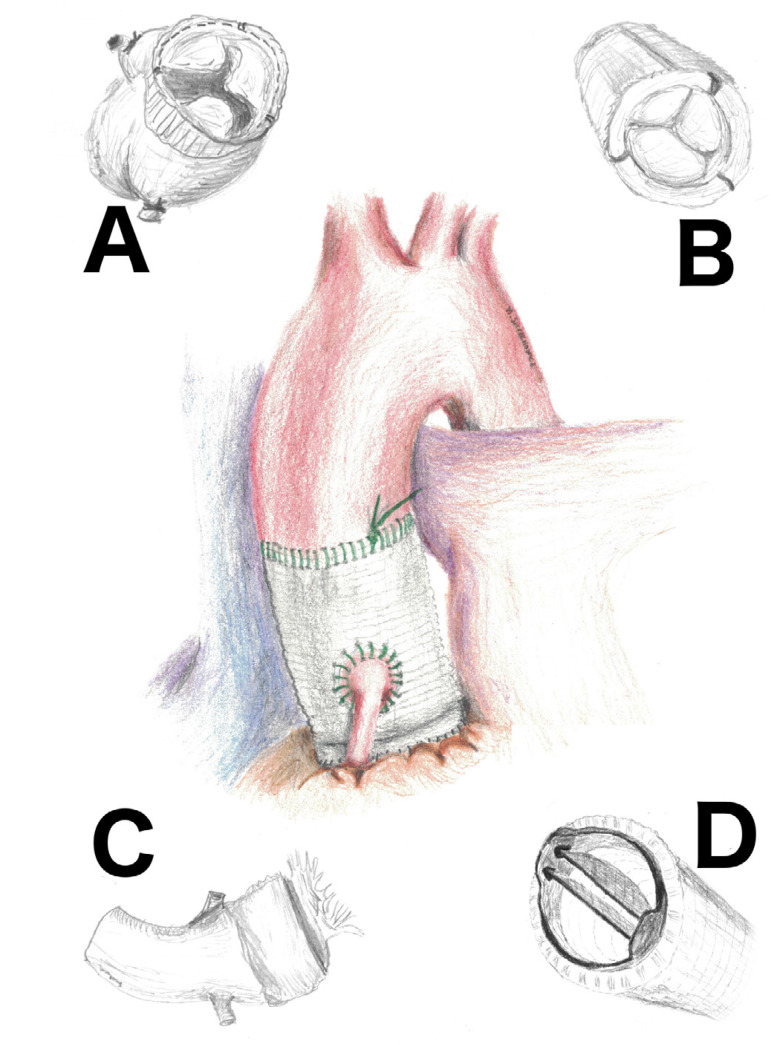
Analyzed options for aortic root replacement (in the middle) due to destructive endocarditis. A) Stentless porcine xenograft, B) biological valve conduit, C) homograft, D) mechanical valve conduit.

In patients with destructive aortic root endocarditis, we predominantly use the stentless porcine xenograft (Freestyle) as a full root replacement, but we also use homograft and conduit valves (Figure 1). The aim of this study was to compare the surgical short- and long-term results (survival and complications within the follow-up) of these solutions in patients with the severe destructive form of aortic valve endocarditis.

## METHODS

### Patient Selection

A total of 483 patients underwent aortic valve and root surgery due to IE in our institution from 1999 to 2018. For 80 (16.6%) of them, root replacement was performed, and they were retrospectively included in our study. In these analyzed cases, aortic root replacement had to be performed due to severe root destruction (infected fistula, large abscess which could not be simply removed, inflammatory aortic aneurysm, or chronic dissection) or extensive infection of the prosthetic aortic valve (detachment of the prosthetic valve ring, atrioventricular dehiscence, or infection of whole prosthetic aortic root). There were no exclusion criteria. We analyzed the patients’ preoperative characteristics, surgical data, and postoperative courses. To obtain data for survival analysis, we performed a follow-up evaluation. Within our sample, we created, analyzed, and compared four groups: in group 1, patients received the Freestyle stentless prostheses (n=53); in group 2, patients received stented bioroots (n=13); in group 3, patients received homografts (n=9); and in group 4, patients received mechanical conduits (n=5). All these groups were considered as independent samples.

### Statistical Analysis

Qualitative data are shown as absolute values and percentages. We compared the distributions between the groups with the chisquared test. Quantitative data are presented as median values with quartiles. We assumed non-normal distributions in all cases because of the relatively low number of cases. To compare the distributions of such data between the groups, we performed the Kruskal-Wallis test. To compare the distributions of quantitative variables between two groups (one group *vs*. all others), the Mann-Whitney U test was used. Survival and freedom from combined endpoint (FFCE), which was defined as death, stroke, aortic valve reinfection, and/or aortic valve reoperation for any cause, were analyzed with the Kaplan-Meier method and general and pairwise group comparisons were performed with the use of the log-rank test. The incidence rates of aortic valve reoperation and FFCE during follow-up were compared with the use of the polynomial multiplication method and are presented as number of events/100 patient-years. Overall, we considered *P*-values < 0.05 as statistically significant. For the statistical analysis, we used the R software v.3.4.3 (R Foundation for Statistical Computing, Vienna, Austria) as well as the IBM SPSS Statistics software, version 25 (IBM Corp.).

## RESULTS

### Preoperative Characteristics

All 80 patients underwent urgent or emergency surgery. Eleven (13.8%) were female and the median age at the time of surgery was 64 years. Forty-nine (61.3%) patients had already undergone diverse types of aortic valve replacement and 10 (12.5%) of them had also had coronary bypasses. The average time from the first aortic valve replacement to the development of IE was 4.4 (1.5 to 7.8) years. Early PVE, which was defined as PVE that occurred within the first year after the valve replacement, was the surgical indication in eight (10%) of our patients (17% of all PVE patients). Additive European System for Cardiac Operative Risk Evaluation, or EuroSCORE, scores were similar in all subgroups (median=19; 17 to 21). Patients’ demographic data and preoperative characteristics are specified in detail in Table 1.

**Table 1:** Preoperative characteristics and comorbidities.

Characteristics	Freestyle	Other biological conduit	Homograft	Mechanical conduit	*P*-value
N	53 (66.25%)	13 (16.25%)	9 (11.25%)	5 (6.25%)	-
Females	7 (13.2%)	1 (7.7%)	3 (33.3%)	0	0.248
Age (years)	70 (62 to 75)	55 (45 to 68)	66 (46 to 70)	55 (48 to 58)	0.124
Ejection fraction	60 (55 to 65)	55 (38 to 63)	65 (60 to 67)	55 (35 to 73)	0.178
Previous heart surgery	33 (62.3%)	8 (61.5%)	8 (88.9%)	3 (60%)	0.465
Aortic valve surgery	31 (58.5%)	8 (61.5%)	7 (77.8%)	3 (60%)	0.751
Mechanical aortic valve prosthesis	11 (20.8%)	4 (30.8%)	5 (55.6%)	2 (40%)	0.156
Mechanical aortic valve conduit	2 (3.8%)	2 (15.4%)	2 (2.2%)	0	0.477
Biological aortic valve prosthesis	20 (37.7%)	4 (30.8%)	2 (22.2%)	1 (20%)	0.709
Biological aortic root prosthesis (all patients had had Freestyle)	4 (7.5%)	2 (15.4%)	1 (11.1%)	0	0.716
Coronary artery bypass grafting	7 (13.2%)	1 (7.7%)	2 (22.2%)	0	0.617
Relevant coronary artery disease	12 (22.6%)	2 (15.4%)	2 (22.2%)	0	0.641
Atrial fibrillation	9 (17%)	0	0	2 (20%)	0.076
Arterial hypertension	25 (47.2%)	7 (53.8%)	3 (33.3%)	1 (20%)	0.518
Chronic kidney disease	13 (24.5%)	4 (30.8%)	3 (33.3%)	3 (60%)	0.397
Diabetes mellitus	3 (5.7%)	4 (30.8%)	0	0	0.020
Additive EuroSCORE	19 (17 to 22)	19 (16 to 20)	19 (17 to 21)	18 (17 to 20)	0.284

EuroSCORE = European System for Cardiac Operative Risk Evaluation

### Surgical Data

All patients were operated upon through median sternotomy, with the use of cardiopulmonary bypass, and all received crystalloid cardioplegia. Severe periannular complications were seen in all cases. In seven cases, there was no definable destructed area, but the poor quality of the very fragile, infected aortic ring and root tissue made conventional aortic valve replacement impossible. Therefore, aortic root replacement was also performed in these cases. Detailed surgical data are listed in Table 2.

**Table 2:** Surgical data.

Characteristics	Freestyle	Other biological conduit	Homograft	Mechanical conduit	*P*-value
N	53 (66.25%)	13 (16.25%)	9 (11.25%)	5 (6.25%)	-
Surgery time (min)	265 (195 to 343)	322 (225 to 478)	398 (245 to 473)	305 (202 to 464)	0.086
Cardiopulmonary bypass time (min)	164 (121 to 229)	210 (114 to 289)	225 (150 to 341)	199 (131 to 316)	0.157
Cross-clamp time (min)	114 (85 to 140)	134 (87 to 146)	146 (114 to 198)	128 (101 to 149)	0.137
Aortic anulus diameter (mm)	25 (25 to 27)	25 (25 to 29)	23 (23 to 27)	27 (23 to 29)	0.396
Periannular complications					
Fistula	4 (7.5%)	1 (7.7%)	2 (22.2%)	2 (40%)	0.106
Abscess	23 (43.3%)	6 (46.2%)	6 (66.7%)	2 (40%)	0.623
Aortoventricular dehiscence	12 (22.6%)	3 (23.1%)	3 (33.3%)	2 (40%)	0.773
Chronic type-A aortic dissection	1 (1.9%)	0	1 (11.1%)	0	0.350
Inflammatory aneurysm of the aortic root	2 (3.8%)	0	1 (11.1%)	2 (40%)	0.009
Concomitant procedures	25 (47.2%)	6 (46.2%)	8 (88.9%)	3 (60%)	0.127
Mitral valve surgery	7 (13.2%)	1 (7.7%)	4 (44.4%)	2 (40%)	0.050
Replacement of the ascending aorta	11 (20.8%)	0	4 (44.4%)	0	0.042
Coronary artery bypass grafting	8 (15.1%)	6 (46.2%)	2 (22.2%)	1 (20%)	0.110
Bail-out bypass	3 (5.7%)	4 (30.8%)	1(11.1%)	1 (20%)	0.072
Circulatory support					
Extracorporeal membrane oxygenation	3 (5.7%)	0	0	1 (20%)	0.311
Intra-aortic balloon pump	7 (%)	3 (%)	0	1 (20%)	0.463

### Postoperative Course

Inotropes or vasopressors were postoperatively used in all cases. The length of mechanical ventilation, intensive care unit length of stay, and hospital length of stay values were similar in all groups (*P*=0.384, *P*=0.658, and *P*=0.620, respectively). There were no significant differences between the analyzed groups regarding postoperative transfusions (*P*=0.280).

Respiratory failure, defined as the need for mechanical ventilation for > 48 hours or the need for continuous oxygen supply > 5 liters/minute for > 24 hours after the extubation despite optimal respiratory therapy, occurred in 22 (27.5%) patients and was equally distributed in all the groups (*P*=0.243).

Acute kidney injury, defined as an increase of serum creatinine level over 1.5 times or reduced urine output (< 0.5 milliliters/ kilogram/hour) over at least six hours, was observed in 16 (20%) patients, with a similar incidence in all the groups (*P*=0.699). Four (5%) patients required a temporary dialysis and two of them (2.5%) died postoperatively. However, no one required dialysis at the moment of hospital discharge. Two patients with mechanical conduits died one day after their operations from severe bleeding due to coagulopathy. The 30-day mortality rate differed between the samples with borderline-significance (*P*=0.055); however, the distinct trends cannot be overlooked here. The early mortality within the Freestyle group was lower than in the mechanical conduit group (15.1% *vs*. 60%). The 30-day mortality rate was much lower in NVE patients than in PVE patients (6.5%, n=2 *vs*. 32.7%, n=16, respectively; *P*=0.006). Detailed postoperative data and the incidences of postoperative adverse events are presented in Table 3.

**Table 3:** Postoperative characteristics.

Characteristics	Freestyle	Other biological conduit	Homograft	Mechanical conduit	*P*-value
N	53 (66.25%)	13 (16.25%)	9 (11.25%)	5 (6.25%)	-
Acute kidney injury	10 (18.9%)	4 (30.8%)	1 (11.1%)	1 (20%)	0.699
Temporary dialysis	3 (5.7%)	1 (7.7%)	0	0	0.805
Atrial fibrillation	12 (22.6%)	5 (38.5%)	1 (11.1%)	0	0.259
Revision due to mediastinal bleeding	7 (13.2%)	4 (30.8%)	0	2 (40%)	0.103
Inferior pericardiotomy due to pericardial tamponade	3 (5.7%)	1 (7.7%)	0	1 (20%)	0.514
Delirium	5 (9.4%)	0	0	0	0.437
Stroke	1 (1.9%)	1 (7.7%)	0	0	0.598
Respiratory failure	17 (32.1%)	4 (30.8%)	0	1 (20%)	0.243
Cardiopulmonary resuscitation	0	1 (7.7%)	1 (11.1%)	0	0.129
Low output syndrome	3 (5.7%)	0	0	1 (20%)	0.311
Pacemaker implantation	7 (13.2%)	1 (7.7%)	0	0	0.524
Packed red cells transfusion (ml)	1200 (600 to 3000)	2700 (600 to 10275)	2400 (800 to 4200)	600 (0 to 3000)	0.280
Fresh frozen plasma transfusion (ml)	900 (0 to 2250)	2400 (600 to 9600)	500 (0 to 2050)	0 (0 to 2400)	0.088
Mechanical ventilation (hours)	14 (2 to 74)	22 (14 to 105)	18 (8 to 90)	30 (24 to 30)	0.384
Intensive care unit length of stay (days)	3 (2 to 10)	3 (1 to 8)	3 (2 to 14)	2 (1 to 16)	0.658
Hospital length of stay (days)	10 (6 to 17)	9 (4 to 12)	7 (7 to 23)	8 (3 to 19)	0.620

### Follow-up

**Fig. 2:**
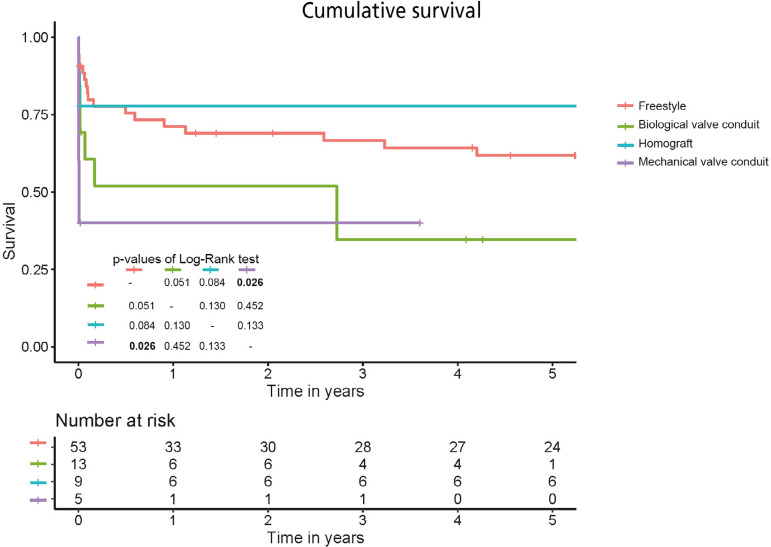
Survival curves for all four analyzed groups.

The median follow-up time was 4.9 (0.8 to 8.9) years. The follow-up data and long-term outcomes are presented in Table 4. The cumulative survival and comparison of the analyzed groups using the log-rank test are presented in Figure 2. Overall, the groups differed from each other (*P*=0.014). There was a significant survival difference between patients who received the Freestyle prosthesis and those who received a mechanical valve conduit (*P*=0.026). There was also a borderline difference between the Freestyle and biological valve conduit patients (*P*=0.051). All other differences were not statistically significant, but there are recognizable trends that correspond to the mean survival times. However, a comparison of each group with all the others together suggests that the worst outcomes occurred after mechanical valve conduit implantation (*P*=0.043) and that homografts may result in better outcomes, compared to all the other options (*P*=0.050). Two of five (40%) patients that underwent mechanical conduit implantation died postoperatively from bleeding and one (20%) died from multi-organ failure. If the first postoperative month is eliminated from the analysis, the Kaplan-Meyer curves look similar and the log-rank test no longer shows significant differences (*P*=0.365).

**Table 4:** Follow-up data.

Characteristics	Freestyle	Other biological conduit	Homograft	Mechanical conduit	*P*-value
N	53 (66.25%)	13 (16.25%)	9 (11.25%)	5 (6.25%)	-
30-day mortality	8 (15.1%)	5 (38.5%)	2 (22.2%)	3 (60%)	0.055
Mean survival with 95% confidence interval (years)	8.1 (6.2 to 9.9)	2.8 (1.1 to 4.4)	13.7 (8.9 to 18.5)	1.4 (0 to 3)	0.014
Aortic valve redo-surgery within the follow-up, absolute values	1 (1.9%)	2 (15.4%)	3 (33.3%)	0	0.005
Aortic valve redo-surgery within the follow-up (events/100 patient-years)	0.4	8	4.3	0	< 0.001
Mean freedom from composite endpoint (death, stroke, aortic valve redo surgery) with 95% confidence interval (years)	7.6 (5.8 to 9.5)	2.1 (0.6 to 3.7)	11.3 (6.3 to 16.4)	1.4 (0 to 3)	0.011
Incidence of composite endpoint within the follow-up (death, stroke, aortic valve redo surgery) (events/100 patient-years)	10.8	44.4	7.4	82.5	< 0.001

**Fig. 3:**
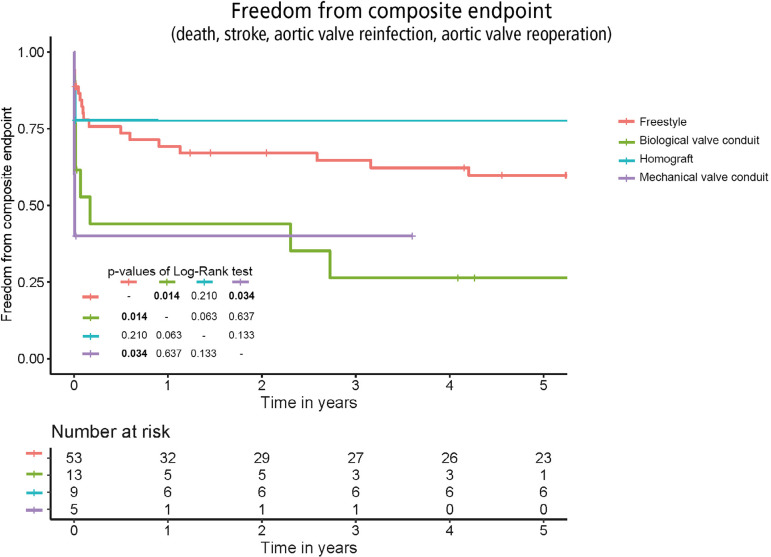
Cumulative freedom from composite endpoint for all four surgical options.

Figure 3 shows the comparison of FFCE for all four samples using the log-rank test. Overall, the outcomes differed between the groups (*P*=0.011). The Freestyle prosthesis had better combined outcomes than the mechanical (*P*=0.034) and biological (*P*=0.014) valve conduits. The advantage of the homograft valve over the conduits was unclear (*P*=0.064). The biological valve conduit had the worst possible outcome if compared to all the other options together (*P*=0.020). The incidences of FFCE were similar in the Freestyle and homograft implantation groups (*P*=0.210).

## DISCUSSION

Short-term outcomes in IE patients are generally poor, with the 30-day mortality rate oscillating between 12.2% and 30%^[10]^. In our sample, 22.5% of patients (n=18) died within the first month after the surgery, which corresponds with the values reported in the literature^[11]^. This high mortality rate is the result of the septic nature of IE. The valvular and perivalvular structures are quickly damaged by the infection and often severely destroyed by the time of diagnosis. Cardiac dysfunction and systemic infection lead to low-output status, mixed septic and cardiogenic shock, and organ failure, which significantly reduces survival despite optimal surgical therapy^[12]^.

Forty-nine (61.3%) patients were operated on because of PVE, which is associated with higher mortality than NVE, as demonstrated in our analysis and the literature. Diagnosing PVE is much more challenging than diagnosing NVE, which often delays the implementation of adequate therapy and local complications occur much more frequently in these cases^[12-14]^.

Classically, for treating IE, the aortic valve homograft has been the option of choice. It enables radical debridement of infected tissues and the destroyed left ventricular outflow tract or anterior mitral cusp can be reconstructed with the anterior mitral cusp of the homograft^[15]^. The reinfection rate is very low because there is no foreign material^[16]^. The disadvantages, however, are the homografts’ limited availability in urgent settings and structural valve degeneration with challenging reoperations over the long term^[15,17]^. In our homograft cohort, this relatively high reoperation rate did not worsen the survival rates, which were very advantageous and similar to those of the Freestyle group.

The second option includes mechanical and biological conduit valves, which are usually implanted using the modified Bentall technique. Due to their immediate availability in all sizes and standardized implantation technique, they are commonly used as aortic root replacements not only for endocarditis, but also for other indications^[18]^. In our sample, they had worse outcomes than the other investigated groups. The reoperation rate was the highest in the group with biological valve conduits, indicating that these conduits are probably not the best solution for treating IE because of the large amount of foreign material, which increases the risk of reinfection^[16]^. Also, the hemostatic properties of conduit valves may not be optimal. Two patients with mechanical valve conduits died from postoperative bleeding, which did not happen within the other groups. However, this fact is difficult to interpret due to small sample size.

The stentless porcine aortic root prosthesis contains a smaller amount of artificial material than the conduits, which theoretically should reduce the probability of reinfection. The implantation technique resembles the one used for homografts, but industrial production makes the results more reproducible^[19]^.

Pulmonary valve autografts (Ross operation) can be a safe alternative to all the mentioned strategies in selected patients, but it has been very seldom performed in our institution and will not be considered here^[20]^. In cases of recurrent infection and extremely severe heart damage, radical steps, such as heart transplantation, may be necessary^[21]^.

If the ascending aorta is damaged by inflammation, it must be replaced as well. It is not an issue if a conduit is implanted because the conduit is long enough to place the distal anastomosis at the level of the proximal aortic arch; however, if a homograft or a Freestyle valve is implanted, a short piece of aortic prosthesis between the root prosthesis and the aortic arch may be necessary, as it was in our study for 11 (20.8%) Freestyle patients and four (44.4%) homograft patients^[22]^. The postoperative complications in our sample were distributed in a manner that is typical for endocarditis. High rates of respiratory failure or acute kidney injury are also described by other authors^[14]^.

Extracorporeal membrane oxygenation (ECMO) was used in 5% of cases and intra-aortic balloon pumps (IABPs) were implemented in 13.8% of cases in our study; these numbers are comparable to those from other studies. Belletti et al.^[23]^ described the rates of ECMO and IABP use in endocarditis patients as 6.6% and 11.1%, respectively; however, their research was not limited to patients who received aortic root replacement. Also, the strategy of cardiac support use can vary from one center to another.

The Freestyle valve does not have survival advantage over homografts, but the reoperation rate within the follow-up was lower in Freestyle patients than among homograft patients. Long-term outcomes were significantly beneficial for the Freestyle/homograft cohort in comparison to the valvular conduits. However, 18 patients (22.5%) died within the first 30 days after the surgery. Survival differences between the groups are strongest within the first postoperative month.

Despite the median age at the time of surgery, which was highest among the Freestyle patients (though without statistical significance), the survival rates and incidences of FFCE within the Freestyle sample were better than those in the other groups. The stentless porcine aortic root xenografts are almost as pliable as homografts, which enables complex reconstructions of the inflamed, destroyed aortic anulus^[24]^. They contain smaller amounts of foreign material than traditional valvular conduits and are available quickly and at any time in all sizes; they do not need to be kept frozen^[25]^.

### Limitations

Our sample size allowed for statistical analysis, but we could get more significant results if we had more patients. That said, this study contains one of the biggest homogeneous samples with destructive aortic root IE patients treated with aortic root replacement described in the literature until now. The subgroup with mechanical conduits is relatively small because in our clinic, in cases of IE, such valves are implanted only at the patient’s request. A prospective randomized study is needed to get clearer conclusions about the described surgical options, but such study is very difficult to perform because it would mostly concern emergency cases.

## CONCLUSION

There is no ideal solution for destructive aortic root endocarditis, but the stentless porcine root prosthesis seems to have good outcomes. The homograft valves allow extensive debridement and reconstruction, but their availability is limited, and the reoperation rate is high. These two strategies are, in patients with destructive IE of the aortic root, significantly better than using valvular conduits. Most postoperative deaths occur within the first 30 days after surgery.

**Table:** 

Authors' roles & responsibilities	
MS	Substantial contributions to the conception or design of the work; or the acquisition, analysis, or interpretation of data for the work; drafting the work or revising it critically for important intellectual content; agreement to be accountable for all aspects of the work in ensuring that questions related to the accuracy or integrity of any part of the work are appropriately investigated and resolved; final approval of the version to be published
AW	Substantial contributions to the conception or design of the work; or the acquisition, analysis, or interpretation of data for the work; drafting the work or revising it critically for important intellectual content; agreement to be accountable for all aspects of the work in ensuring that questions related to the accuracy or integrity of any part of the work are appropriately investigated and resolved; final approval of the version to be published
SM	Substantial contributions to the conception or design of the work; or the acquisition, analysis, or interpretation of data for the work; drafting the work or revising it critically for important intellectual content; agreement to be accountable for all aspects of the work in ensuring that questions related to the accuracy or integrity of any part of the work are appropriately investigated and resolved; final approval of the version to be published
AM	Substantial contributions to the conception or design of the work; or the acquisition, analysis, or interpretation of data for the work; drafting the work or revising it critically for important intellectual content; agreement to be accountable for all aspects of the work in ensuring that questions related to the accuracy or integrity of any part of the work are appropriately investigated and resolved; final approval of the version to be published
KZ	Substantial contributions to the conception or design of the work; or the acquisition, analysis, or interpretation of data for the work; drafting the work or revising it critically for important intellectual content; agreement to be accountable for all aspects of the work in ensuring that questions related to the accuracy or integrity of any part of the work are appropriately investigated and resolved; final approval of the version to be published
JE	Substantial contributions to the conception or design of the work; or the acquisition, analysis, or interpretation of data for the work; drafting the work or revising it critically for important intellectual content; agreement to be accountable for all aspects of the work in ensuring that questions related to the accuracy or integrity of any part of the work are appropriately investigated and resolved; final approval of the version to be published
